# Comment on: Platelet counts and mean platelet volume in association with serum magnesium in maintenance hemodialysis patients

**DOI:** 10.12861/jrip.2012.05

**Published:** 2012-01-01

**Authors:** Azar Baradaran

**Affiliations:** ^1^Department of Pathology, Isfahan University of Medical Sciences, Isfahan, Iran

**Keywords:** Chronic kidney disease, Platelets, Magnesium, End-stage renal disease, Hemodialysis

**Implication for health policy/practice/research/medical:**

Bleeding tendency is frequently seen due to disturbances in platelet adhesion and aggregation in hemodialysis. Rafieian-Kopaie and his colleague undertaken a study on 36 chronic hemodialysis patients. They found a negative association of serum magnesium with mean platelet volume. This finding needs further consideration to find clinical significance of this study.

In patients with end-stage kidney disease under hemodialysis, thrombotic disorders and bleeding are major complications (1). Uremic toxins which accumulate in the blood of chronic kidney disease patients may influence platelet function, induce hemostatic disorder and mediate thrombotic disorders (1,2). Bleeding tendency of dialysis patients is regarded as hemorrhagic symptoms and prolongation of bleeding time (3,4). Bleeding tendency of dialysis is multifactorial (3,4). It is evident that abnormal platelet function is a major factor, while bleeding occurs despite a normal coagulation profile or elevated coagulation factor levels and normal platelet number (2-5). Platelet dysfunction may be as a consequence of decreased dense granule content, decreased sensitivity to platelet agonists, abnormal expression of platelet glycoproteins, defective arachidonate metabolism and depressed prostaglandin metabolism and also impaired platelet adhesiveness. Various factors responsible for platelet dysfunction such as, increased nitric oxide production, von Willebrand factor abnormalities, anemia, uremic toxins and the use of some drugs like non-steroidal anti-inflammatory drugs, aspirin (3-6). Activation of platelets and platelet degranulation can occur during the hemodialysis. A part of this activation may also occur due to contact of blood to the roller pump segment and microbubbles may play a role (4-6). Thus bleeding tendency is frequently seen due to disturbances in platelet adhesion and aggregation in hemodialysis (3-6). Rafieian-Kopaie and his colleague undertaken a study on 36 chronic hemodialysis patients (7). They found a negative association of serum magnesium with mean platelet volume. This finding needs further consideration to find clinical significance of this study.

## Author’s contribution

AB is the single author of the manuscript.

## Conflict of interests

The author declared no competing interests.

## Ethical considerations

Ethical issues (including plagiarism, data fabrication, double publication) have been completely observed by the author.

## Funding/Support

None.
